# The study of knowledge management capability and organizational effectiveness in Taiwanese public utility: the mediator role of organizational commitment

**DOI:** 10.1186/s40064-016-3173-6

**Published:** 2016-09-09

**Authors:** Chia-Nan Chiu, Huei-Huang Chen

**Affiliations:** Graduate of Information Management, Tatung University, No. 40, Sec. 3, Zhongshan N. Rd., Taipei City, Taiwan, ROC

**Keywords:** Knowledge management capability, Knowledge infrastructure capability, Knowledge process capability, Organizational effectiveness, Organizational commitment

## Abstract

Many studies on the significance of knowledge management (KM) in the business world have been performed in recent years. Public sector KM is a research area of growing importance. Findings show that few authors specialize in the field and there are several obstacles to developing a cohesive body of literature. In order to examine their effect of the knowledge management capability [which consists of knowledge infrastructure capability (KIC) and knowledge process capability (KPC)] and organizational effectiveness (OE), this study conducted structural equation modeling to test the hypotheses with 302 questionnaires of Taipei Water Department staffs in Taiwan. In exploring the model developed in this study, the findings show that there exists a significant relationship between KPC and OE, while KIC and OE are insignificant. These results are different from earlier findings in the literature. Furthermore, this research proposed organizational commitment (OC) as the mediator role. The findings suggest that only OC has significant mediating effects between KPC and OE, whereas this is not the case for KIC and OE. It is noteworthy that the above findings inspired managers, in addition to construct the knowledge infrastructure more than focus on social media tools on the Internet, which engage knowledge workers in “peer-to-peer” knowledge sharing across organizational and company boundaries. The results are likely to help organizations (particularly public utilities) sharpen their knowledge management strategies. Academic and practical implications were drawn based on the findings.

## Background

### Research background

Currently, many organizations are dependent on applying knowledge management (KM) in addition to successful application of tangible assets and natural resources to achieve high performance (Lee and Sukoco 2007). Many studies on the significance of KM in the business world have been performed in recent years (Metaxiotis et al. 2005). Government organizations, such as public utilities, are now expending significant efforts on technological and technical innovation to increase competitiveness and upgrade their capabilities. It is therefore more interesting to investigate the knowledge management issues in public utilities. In Taiwan, most organizations have realized the growing importance of knowledge management. Therefore, it is necessary to conduct research to understand the level at which organizations are able to implement successful knowledge management practices.

Findings show that few authors specialize in the field and there are several obstacles to developing a cohesive body of literature. Low levels of international cooperation among authors and international comparisons mean that the literature is fragmented (Massaro et al. 2015).

Meanwhile, knowledge workers are now estimated to outnumber all other workers in North America by at least a four to one margin (Haag et al. 2006). Due to the rapid global expansion of information-based transactions and interactions, this situation will become a universal phenomenon. This can also be related with market and research. It could be expected that managing knowledge workers can be a difficult task as most knowledge workers prefer some level of autonomy and do not like being overseen or managed (Bhanu et al. 2016). Managers must be carefully considered before being assigned to a knowledge worker, as their interests and goals will affect the quality of the work.

### Research motivation and purposes

Because the value of KM practices is well recognized around the world, there are limited empirical investigations on the relationships between KMC and organizational effectiveness. A recent study by Gold et al. (2001) shed light on the relationships between KMC and organizational effectiveness. According to their study, KMC can be assessed via two major constructs: the knowledge infrastructure capability (KIC) and knowledge process capability (KPC). The results disclose the positive relationships between KPC and organizational effectiveness and between KIC and organizational effectiveness.

Additionally, as De Angelis (2013) state, the public sector is influenced by a growing need for: “competition, performance standards, monitoring, measurement, flexibility, emphasis on results, customer focus and social control”. However, there are fewer studies focusing on public sector KM than those focusing on KM in the private sector (Oluikpe 2012, Ringel-Bickelmaier and Ringel 2010), even though “KM initiatives have always been integrated in government tasks, inseparable from strategy, planning, consultation, and implementation” (Riege and Lindsay 2006). Most studies on either KM or KMC generally use private organizations as research subjects and rarely perform empirical studies of public utilities. This gap leads to the initial research motivation of this study, which is to consider whether the previously discussed relevant studies can be applied to public utilities.

Next, prior research sheds light on relationships between human resources and organizational effectiveness. For example, although the existence of a proper technology infrastructure is a necessity for KM, the research that examined the link between information technologies and organizational performance indicators has remained inconclusive and has failed to explain a direct relationship between information technology and performance (Emadzade et al. 2012).

Finally, considering that most knowledge workers prefer some level of autonomy and do not like being overseen or managed (Bhanu et al. 2016), research on this topic should include both willingness and motivation. This gap leads to the second research motivation of this study, which is proposing a role for “organizational commitment” to fill this gap.

In this regard, the purpose of this study is to bridge these gaps in the literature by examining the correlation of KMC and organizational effectiveness by choosing a public utility as its research object so as to expand the scope of relevant studies and serve as a reference to scholars in this area in the future.

This study is specifically aimed at exploring the mediating effect of a human capital, namely organizational commitment in the relationship between KMC and organizational effectiveness. An understanding of the current situation and the actual needs of employees can help organizations (particularly public utilities) implement key success factors, sharpen their knowledge management strategies, and improve overall competitiveness and operational performance.

## Literature review and research design

### Relevant literature

To shed light on this subject, researchers have examined the many differences between various perspectives. The descriptions are as follows:

#### Knowledge management (KM) and knowledge management capability (KMC)

Knowledge management is the employment and development of the knowledge assets of an organization to achieve the organizational goals. This knowledge consists of both explicit and implicit knowledge (Theriou and Chatzoglou 2008). Knowledge management involve the creation, manipulation, storage and sharing of knowledge among people in a community of practice. Knowledge management manages the knowledge flows in an organization (Hislop 2013). To enhance organizational performance, knowledge management strategies must be incorporated and implemented so that the organization attains a competitive edge. Organizations that are skilled in knowledge management consider knowledge to be human capital and have developed organizational rules and values to support knowledge production and sharing (Metaxiotis et al. 2005; Meyer et al. 2002).

**Fig. 1:**
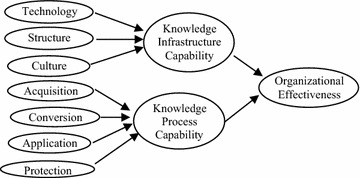
Knowledge management capabilities and organizational effectiveness (Gold et al. 2001)

Knowledge management capability (KMC) is an organizational mechanism to continually and intentionally create knowledge in organizations (Von Krogh et al. 2001). In addition, Gold et al. (2001) proposed knowledge management (KM) infrastructural capabilities and process capabilities as direct determinants of organizational effectiveness (Fig. 1). They argued that an organization must leverage its existing knowledge management capabilities and apply the knowledge in its operations to sustain competitiveness.

With regard to previous research, KMC is divided into two categories: knowledge infrastructure capabilities and knowledge process capabilities (Gold et al. 2001; Lee and Sukoco 2007; Aujirapongpan et al. 2010; Miils and Smith 2011; Smith et al. 2010). This paper applies the Gold et al. (2001) model for these two capabilities.

#### Knowledge infrastructure capability (KIC)

Knowledge infrastructure capabilities (KIC) are required to build and maintain generic capabilities that are shared with organizational activities and functions. According to the study by Gold et al. (2001), knowledge infrastructure capabilities can be assessed through three major constructs: structural infrastructure, technical infrastructure, and cultural infrastructure. This study adopted items to measure the three constructs of knowledge infrastructure capability; the descriptions are as follows.

- Structure

Structural infrastructure refers to the physical layout and organization hierarchy (Armbrecht et al. 2001). A proper physical structure, such as office design and office locations, is favorable for knowledge sharing. Flexible hierarchical structures, such as matrix teams or flattened organizations, can also increase communication with individuals and sharing behavior within the organization (Gold et al. 2001; Armbrecht et al. 2001). Structural infrastructure refers to the physical layout and organization hierarchy (Armbrecht et al. 2001). A proper physical structure, such as office design and office locations, is favorable for knowledge sharing. Flexible hierarchical structures, such as matrix teams or flattened organizations, can also increase communication with individuals and sharing behavior within the organization (Gold et al. 2001; Armbrecht et al. 2001). Enterprises can establish strategies to form a knowledge sharing culture, which creates a desire for knowledge among their employees that keeps the enterprises themselves steady with regard to the continual application, distribution, and creation of knowledge (Hauschild et al. 2001).

b. Information technology

Gold et al. (2001) stated that technology refers to the crucial element of the structural dimension needed to mobilize social capital for the creation of knowledge. Moreover, they identified technological dimensions as those that are part of effective knowledge management, including business intelligence, collaboration, distributed learning, knowledge discovery, knowledge mapping, opportunity generation, and security. Information technology is often cited in the literature as an important KM infrastructural capability, enabling or supporting core knowledge activities such as knowledge creation, knowledge distribution and knowledge application (Gold et al. 2001). From the KM perspective, the technical knowledge management capability can assist firms in enabling the rapid acquisition, storage, and exchange of knowledge, mapping internal or external knowledge sources, integrating organizational knowledge flows, and applying existing knowledge to create new knowledge (Chuang 2004; Gold et al. 2001). Therefore, the technical knowledge capability, that is, the ability to integrate and deploy knowledge by using information communication technology (ICT) effectively, is an essential attribute in a knowledge organization. In developing effective knowledge management, it is important to understand the stages of ICT and fundamental issues and factors affecting the adoption or rejection of technologies. Employees need to have their disposal tools that improve their capacity to share knowledge with colleagues wherever and whenever. These technologies enhance knowledge management and usually involve more people in knowledge creation process as they allow multiple people to collaborate when creating knowledge (Majchrzak et al. 2013).

c. Culture

Gold et al. (2001) argued that culture is the supportive capability for the valuation of organizational knowledge and builds an interactive, collaborative atmosphere among the organization’s members. The organizational culture is considered a complicated set of values, beliefs, behaviors, and symbols affecting the knowledge management in organizations (Ho 2009). Thus, a friendly knowledge culture is regarded as the main factor that influences knowledge management and the application of its outcomes (Miils and Smith 2011). Sin and Tse (2000) concluded that organizational culture values such as consumer orientation, service quality, informality, and innovation are significantly related to organizational performance.

Moreover, the failure of many knowledge transfer systems is often a result of cultural factors rather than technological oversights (Pirkkalainen and Pawlowski 2013). For this reason, organizational culture is a major barrier to success in the KM.

#### Knowledge process capability (KPC)

KM is a dynamic and continuous set of processes and practices embedded in individuals as well as in group and physical structures. At any point in time in a given organization, individuals and groups may be involved in different aspects of the KM process (Pirkkalainen and Pawlowski 2014). Thus, KM must be considered as a sequence of activities and events (i.e. creation, storage, transfer or application of knowledge) that ultimately lead to KM outcomes (Eaves 2014).

KPC consists of organizational capabilities that manipulate knowledge stored in the form of standard operating procedures and routines throughout the organization. Edvission (2000) suggests that KPC consists of four steps: sharing tacit knowledge, creating concepts, justifying concepts, and facilitating cross-leveling knowledge. Gold et al. (2001) offer another four-stage KPC model including acquisition, transformation, application, and protection by grouping processes from other empirical studies. Alavi and Tiwana (2003) investigate the KM process framework that consists of four stages: creation, storage/retrieval, transfer, and application. Cui et al. (2005) also mentioned that KM capabilities consist of three interrelated processes: acquisition, conversion, and application. Knowledge is not only an important resource for an organization but also serves as a basic source of competitive advantages. Therefore, KM capabilities refer to the KM processes in an organization that develop and use knowledge within the firm (Gold et al. 2001). From Gold et al. (2001) and Cui et al. (2005), Liao and Wu (2010) comprehensively examined knowledge management activities from the perspective of organizational capabilities. They argue that there are three main processes: acquisition, transformation, and application. Although there are still many classifications of KM, this study addresses the viewpoints of organizational capabilities and focuses on these three dimensions. The descriptions are as follows.

- Acquisition

Acquisition is concerned with seeking knowledge outside the organization and creating new knowledge from the interaction between new knowledge and previous knowledge in the organization. Thus, the new knowledge will benefit innovation development and organizational effectiveness. Acquisition refers to the ability of an organization to identify access and collect the internal and external knowledge that is necessary for its activities (Gold et al. 2001; Zahra and George 2002). Knowledge acquisition results from individual participation and interactions between tasks, technologies, resources and people within a particular context (Anha et al. 2006). The knowledge which is externalized and captured by people who need it can increase the productivity and profitability of firms (Mtega et al. 2013).

b. Transformation

Knowledge transfermation is an important process of KM in organizational settings and refers to the transfer of knowledge to locations where it is needed and can be used. Organizational must carefully transform aspects of tacit knowledge into explicit knowledge; otherwise, the tacit knowledge may be lost (Gold et al. 2001; Pirkkalainen and Pawlowski 2013).

Transformation is the ability for enterprises to transform knowledge to be assimilated or accessible within the organization (Gold et al. 2001). If enterprises can transform tacit knowledge into explicit and codified knowledge, enterprises would utilize the more explicit knowledge efficiently and effectively to innovate or perform better (Egbu 2004). Effective usage of the knowledge in business requires the transformation of acquired knowledge from internal and external resources to organizational knowledge. These transformations, which occur along with the supply of data, information and knowledge cycle, are transient and must transform data into information and transform information into organizational knowledge to maximize the benefits of this process (Bhatt 2001).

c. Application

Application is the knowledge use process. Process characteristics that have been associated with the application of knowledge include storage, retrieval, application, contribution and sharing (Gold et al. 2001). The application process is defined as the way knowledge is used within the organization. Processes such as sharing or distributing knowledge would be important for knowledge management (Carrillo et al. 2004). With the assistance of information technology such as an intranet, database systems, or non-information technology tools such as brainstorming sessions and research collaboration, enterprises can exploit the knowledge within the organizations (Carrillo et al. 2004). Therefore, enterprises can increase performance and innovation.

Knowledge application involves activities that show that the organization is applying its knowledge (Bhatt 2002). Moreover, knowledge application means activating knowledge to create value in the organization, which can be reflected in innovations, creations and new products (Miils and Smith 2011). Dröge et al. (2003) believed that companies will be successful in creating a competitive advantage in the long run if they produce knowledge with lower cost and higher speed compared to competitors and apply it effectively and efficiently.

#### Organizational commitment

Organizational commitment has received substantial attention in past studies due to its significant impact on work attitudes such as job satisfaction, performance, absenteeism, and turnover intentions. Paul and Anantharaman (2004) found in their study of information technology companies in India that of all the HRM variables that correlate with commitment.Organizations are constantly engaged in devising employment practices to retain employees and induce in them higher levels of commitment (Hislop 2013).

Different scholars have defined organizational commitment depending on their backgrounds. The most significant ones belong to Meyer et al. (2002), they suggested different kinds of commitment as following sentences:

- Affective commitment

It refers to employees’ emotional concern about organization, their sense of solidarity with organization, and their active presence in it. Usually, employees who possess organizational commitment are willing to remain in organization and this is one of their desires.

b. Normative commitment

It refers to employees’ obligation to remain in organization. Therefore, employees will remain in organization until they believe that remaining in organization is appropriate and accurate based on their opinion.

c. Continuous commitment

This kind of commitment is about costs and benefits which are related to remaining in or quitting organization. In fact, this commitment suggests a kind of calculation which is referred to as rational commitment and expresses that quitting organization will have exorbitant expenditures for employees.

Moreover, Govindasamy and Jayasingam (2009) noted that organizations wanting to retain knowledge workers and expecting them to develop stronger organizational commitment should encourage knowledge sharing among employees in various ways, including providing organizational support, establishing policies that create a supportive environment for knowledge sharing, promoting knowledge sharing activities, encouraging teamwork among employees and forging close relationships between members of the management team and the employees (Benson and Brown 2007). Govindasamy and Jayasingam (2009) indicated that the willingness of workers to share their knowledge may influence the organizational commitment level.

#### Organizational effectiveness

The effects of knowledge activities on performance are shown in a wide range of domains, and the broadest concept reflects performance in the research on strategic management and organization theory. Organizational effectiveness, including multiple criteria or predictors, for example, profitability (Tippins and Sohi 2003), operational efficacy, and market share (Choi and Lee 2002), is ordinarily referred to as the level at which a firm achieves its strategic goals. Organizational effectiveness includes the outcome of knowledge management capabilities, such as improved coordination of effects, the rapid commercialization of new products, the ability to anticipate surprises, and responsiveness to market changes (Gold et al. 2001). With greater knowledge or practices of infrastructures and process capabilities, the organization can operate well in knowledge management.

*The competing values framework* The competing values framework (CVF) is one of the most influential and extensively used models in the area of organizational culture research. The four effectiveness criteria models in the CVF are also called four organizational culture types. Based on former organizational culture studies in the literature, Cameron and Quinn (2006) termed the four culture types as Clan, Adhocracy, Market, and Hierarchy, respectively. CVF does not attempt to explore the panorama of organizational culture. Rather, it looks at the value dimensions related to effectiveness. Moreover, this model can integrate most organizational culture dimensions proposed in the literature.

**Fig. 2:**
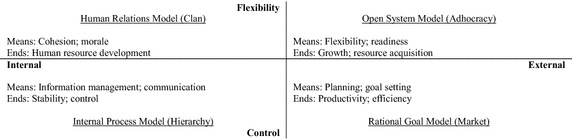
Competing values framework (CVF) (Cameron and Quinn 2006)

Cameron and Quinn (2006) argued that CVF is one method and mechanism designed to help organizations diagnose and make proper changes to organizational culture that will improve execution of a new company-wide direction. CVF is characterized by a two-dimensional space that reflects different value orientations, as shown in Fig. 2.

The first dimension in this framework, the flexibility-control axis, shows the degree to which the organization emphasizes change or stability. The second dimension in this framework, the internal-external axis, addresses the organization’s choice between focusing on activities occurring within the organization (internal) and those occurring outside the organization (external). The two dimensions of the CVF classify four human relations models (human relations model, open system model, rational goal model, and internal process model), each containing a different set of effectiveness criteria.

This study applies the CVF to analyze the relationship between the KMC and organizational effectiveness. The reasons for choosing this framework are as follows.

First, the CVF emerged as a result of empirical research on the question of what makes organizations effective (Ubius and Alas 2009). Second, a large amount of empirical studies have established the reliability and validity of the CVF (Ralston and Terpstra-Tong 2006).

### Research hypotheses

#### Interrelationship between knowledge infrastructure capability and organizational effectiveness

As a basic system, infrastructure is fundamental to organizational activities. Longman and Mullins (2004) suggested that a proper organization structure influences the success of project implementation. In organizations, synergies result from combining infrastructure capabilities and other organizational resources (Melville et al. 2004). Infrastructure is required to build and maintain organizational capabilities and share capabilities with other functions within and across organizations. KIC are capabilities that are essential to support organizational activities by coordinating and controlling strategies among divisions and business units. Moreover, the previous research, e.g., Gold et al. (2001), Lee and Choi (2003), Gosh and Scott (2007), Zack et al. (2009), Emadzade et al. (2012), has shown that KMC affects organizational performance.

Based on the foregoing information, this study proposes the following hypothesis:

##### **Hypothesis 1**

Knowledge infrastructure capability has a significant positive effect on organizational effectiveness

#### Interrelationship between knowledge process capability (KPC) and organizational effectiveness

The knowledge management processes are in the literature mentioned as the knowledge management practices. It is an interrelated set of various business processes developed in an organization to create, store, transfer, and apply the knowledge. Knowledge management practices the first stage is knowledge acquisition, knowledge creation, knowledge storage, knowledge distribution, knowledge use, and knowledge maintaining (Patrick and Sonia 2009). Knowledge process capability improves organizational processes such as innovation, collaborative decision-making and individual and collective learning (King 2009).

KPC are believed to contribute positively to organizational effectiveness by enabling individuals to effectively exploit existing knowledge and explore new knowledge. KPC have been considered an important antecedent for overall organizational effectiveness (Gold et al. 2001). Holsapple and Joshi (2002) introduced five activities of the knowledge chain to realize KPC in an organization: knowledge acquisition, generation, selection, assimilation, and emission.

In summary, the result of efficiently managed KPC is believed to enhance organizational effectiveness. Based on the studies noted above, this study proposes the following hypothesis:

##### **Hypothesis 2**

Knowledge process capability has a significant positive effect on organizational effectiveness

#### Interrelationship between knowledge management capability (KMC) and organizational commitment

Nowadays, it is completely accepted that human resource is the main element in knowledge management productivity (Zahedi and Tejari 2008). Many empirical research results have showed that KM have great influence on organizational outcomes in terms of innovation, product quality, and improvement of employees morale (Alzoubi and Alnajjar 2010; Sireteanu and Grigoruta 2007; Pentland 2003). Creation of a favorable work environment and securing high levels of trust among employees and employer-employee relationships are crucial factors in knowledge sharing (Kurtoğlu 2007). In order to avoid losing the qualified employees or to minimize prospective loss of leaving employees organizations must transform the individual knowledge possessed by the employees into organizational knowledge. Rendering organizational commitment among employees is one of the most important ways. Alzoubi and Alnajjar (2010) studied KM architecture tsted a set of variables related to Knowledge management revealed that the pillars of knowledge management architecture consist of strategy and commitment, information systems, culture, and communication. Knowledge management requires a major shift and commitment of everyone in the oranization in adopting each factor of knowledge management to make it works (Gupta et al. 2000). Working together as a team on various projects has developed a good culture and commitment among auditors that encourage knowledge application and dissemination.

Many scholars have conducted research on the relationships between KM and human resource management (HRM). In turn, Phillips (2011) found that KM can influence an employee’s perception of quality. Chen (2009) found that knowledge sharing and job satisfaction are significantly and positively correlated. Moreover, Govindasamy and Jayasingam (2009) noted that organizations wanting to retain knowledge workers and expecting them to develop stronger organizational commitment should encourage knowledge sharing among employees through organizational support, policies that create a supportive environment for knowledge sharing, promoting knowledge sharing activities, and encouraging teamwork among employees and close relationships between members of the management team and the employees. Govindasamy and Jayasingam (2009) indicated that the willingness of workers to share their knowledge may influence the organizational commitment level. As can be inferred organizational commitment is key to ensuring continuance and knowledge sharing.

In summary, the result of KMC is believed to impact organizational commitment. Based on the studies noted above, this study proposes the following hypotheses:

##### **Hypothesis 3**

Knowledge infrastructure capabilities have significant positive effects on organizational commitment

##### **Hypothesis 4**

Knowledge process capabilities have significant positive effects on organizational commitment

#### Interrelationship between organizational commitment and organizational effectiveness

Organizational commitment is a critical construct for any organization to succeed. Employee commitment is seen as the key factor in achieving competitive performance (Sahnawaz and Juyal 2006). Meyer et al. (2002) defined commitment as a force that binds an individual to a course of action that is of relevance to a particular target. When employees, as noted by Okpara (2004) and Warsi et al. (2009), believe that they will grow and learn with their current employers, their level of commitment to stay with that particular organization is higher. To allow employees to improve their job efficiency, there is a significant need for strong and effective human resource strategies. These strategies must enhance employees’ commitment to their career and organization, reduce turnover intentions and make organizational politics favorable to all employees. If the employees do not understand the company culture, cannot fit in or lack a sense of identification, they will choose to leave their organization (Autry and Daugherty 2003).

Demirel (2008) in his/her study explained organizational commitment by demonstrating its potential consequences according to which organizational commitment is “The individual’s contribution to the organization. It comprises of contributions such as enhancing organizational performance, resolving absenteeism and reduction of worker turnover rate. As the level of commitment to the organization rises so does the level of effort for the organization”. Moreover, several researchers argued that the organizational performance and growth are dependent on successful Human resource development management in terms of enhancing motivation, performance, involvement loyalty and commitment (Sharabi and Harpaz 2010).

According to the statement above, because not all employees are equally willing to provide constructive input and feedback to organizations, this study assumes that organizational commitment has mediating effects on organizational effectiveness. Thus, the study proposes the following hypotheses:

##### **Hypothesis 5**

Organizational commitment has a significant positive effect on organizational effectiveness

##### **Hypothesis 5a**

Organizational commitment has a significant mediating effect between the knowledge infrastructure capability and organizational effectiveness.

##### **Hypothesis 5b**

Organizational commitment has a significant mediating effect between the knowledge process capability and organizational effectiveness.

### Research model

**Fig. 3:**
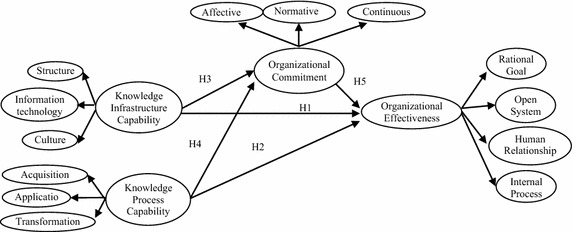
Research model

According to Gold et al. (2001), this study argues that KIC and KPC are antecedents of organizational commitment. Additionally, organizational commitment supports, assists, and facilitates organizational effectiveness. To support the proposition, this study employs a mediating model by positioning organizational commitment as a mediator between KIC/KPC and organizational effectiveness. Based on the correlations observed in the relevant literature, this study established the research framework shown in Fig. 3. Among these variables, KIC and KPC are predictor variables, organizational commitment is a mediating variable, and organizational effectiveness is an outcome variable. This study considered whether significant correlations exist among these variables.

Although many studies have been done in the area of KMC and organizational effectiveness, to the knowledge of the authors, the relationship between KMC and organizational commitment as well as the mediating role of organizational commitment has not been adequately explored hitherto, also no previous studies has examined both of them empirically.

### Definitions and measurement of variables

In this study, four major sections are operationalized: (1) knowledge infrastructure capability, (2) knowledge process capability, (3) organizational commitment, and (4) organizational effectiveness. A survey questionnaire will be designed for this study. The operational definition, measured variables, and sources of the measured dimensions in this study’s questionnaire are illustrated in Table 1. The variables are measured using a 5-point Likert scale, with 1 denoting strong disagreement and 5 denoting strong agreement.

**Table 1:** Operational definition, measured variables, and sources of the measured dimensions

Dimensions	Operational definition	Variables	Source	Measure scale
Knowledge infrastructure capability (KIC)	Organizational capabilities to support knowledge activities in organizations, including structure, information technology and culture	Structure Information Technology Culture	De Long and Fahey (2000) Hanley and Dawson (2000) Alavi and Leidner (2001)	5-point Likert scale measure questionnaire
Knowledge process capability (KPC)	Organizational capabilities to manipulate knowledge that are stored in the form of standard operating procedures and routines throughout the organization, including acquisition, transformation and application	Acquisition Transformation Application	Gold et al. (2001) Lee and Choi (2003) Chuang (2004)	
Organizational commitment	The relative strength of the identification of the individual and his involvement with his particular organization, including affective, normative and continuous	Affective Normative Continuous	Meyer et al. (2002) Autry and Daugherty (2003) Hakanen et al. (2006)	
Organizational effectiveness	The level at which a firm achieves its strategic goals, including rational goals, open system, human relationships and internal process	Rational goal Open system Human relationship Internal process	Cameron and Quinn (2006) Ubius and Alas (2009) Ralston and Terpstra-Tong (2006)	

### Study method

The case study method is highly effective when there is only limited knowledge about the phenomenon and when the purpose of the research is to generate a framework of knowledge to facilitate understanding how the problems can be solved. The goal of the fundamental research is to enable an understanding of the processes and introducing new theoretical relationships. A single-case study can also be used, aside from explanatory purposes, to pursue exploratory goals (Sekaran 2000). The focus of this study was limited to a single public utility (TWD) in a geographically limited area (Taiwan). The situation has a real-life context and possesses a descriptive and exploratory purpose for learning from previous experience, and there have only been a few studies conducted on the issue. In this state, the single-case study has been adopted as the most appropriate research tool.

### Data collection

There were four main stages for the data collection. These stages were implemented to ensure high reliability and validity of the data collection.

#### Research objects

Within knowledge management (KM), the public sector is an important and specific research context. According to Edge (2005), KM has the potential to influence greatly and improve the public sector renewal processes. Indeed, within the public sector, KM is a powerful enabler in the current drive for increased efficiency in all areas (Mcadam and Reid 2000). As Jain and Jeppesen (2013) outline, it is often argued that public sector organizations face greater pressures for representativeness, accountability and responsiveness than private sector firms. Additionally, as De Angelis (2013) state, the public sector is influenced by a growing need for competition, performance standards, monitoring, measurement, flexibility, emphasis on results, customer focus and social control. Public sector practitioners must recognize that their organizations work in a unique context in which their stakeholders and accountability differ significantly from those of the private sector—blindly applying private sector KM tools and models may be counterproductive.

There are two tap water supply systems in Taiwan, namely the Taiwan Water Corporation (TWC) and Taipei Water Department (TWD). Based on information revealed by the official websites of the two companies, at the end of 2012, the total number of people served by TWC was 6.669 million, and the percentage of the population served was 91.81 %. The total number of employees was 5513, and the number of service customers per employee was 1210. In contrast, during the same period, the total number of people served by TWD was 3957 million, the percentage of the population served was 99.6 %, the staff of TWD totaled 1032, and the number of service customers per employee was 3835. According to the service indicators, the latter’s metrics are better than the former; therefore, TWD should be used as a benchmark company.

#### Sampling method

The most common way of obtaining large amounts of data in a relatively short period of time in a cost-effective way is by means of standardized questionnaires. Questionnaire design requires a rigorous process if we want to produce an instrument that yields reliable and valid data and, accordingly, whole volumes have been written on how to construct instruments of good quality (Dörnyei 2010). A survey is used because it has higher generalizability and greater external reliability, as they are based on actual marketing exchanges (Churchill and Iacobucci 2005). In determining the sample size for this study, the sample size selection is based on the criterion set according to Roscoe’s Rule of Thumb (Sekaran 2003). A sample that is larger than 30 and less than 500 is appropriate for most research.

Based on information from the official website of the research object, at the end of 2015, the staff members totaled 622. This research referred to the sample size for the finite population formula. A 95 % degree of confidence level corresponds to d = 0.05 and the sample size required can be calculated according to the following formula. This study needs to sample at least 238 individuals (rounded up).

$${\text{n}} = \frac{N}{{N\left( {\frac{2d}{{Z_{\alpha } /2}}} \right)^{2} + 1}} = 238\quad {\text{N}} = 6 6 2 ,\,{\text{d}} = 0.05,\,{\text{Z}}_{ \propto } /2 = 1.96$$

Note: n = required sample size, N = population size, d = standard deviation, Z = z-score

#### Questionnaire design

The questionnaire in this study was designed primarily from previous studies. Some modifications have been made to fit the current study; as the content was developed in the English version, for considering the Taiwanese respondents, whose main language is Chinese, the questionnaire was translated into Chinese. Afterward, native Taiwanese who were bilingual in Chinese and English translated the Chinese questionnaire back into English to confirm the accuracy of the translation. Any discrepancies found when comparing the two versions were corrected, and thus, consistency between the Chinese and English questionnaires was assured.

The survey includes 78 questions, all of which are measured on five-point Likert-type scales. The scales are anchored by (1) strongly disagree, with (3) neutral (either agree or disagree) as the midpoint, and (5) strongly agree.

When self-report questionnaires are used to collect data simultaneously from the same participants, common method variance (CMV) may be a concern. Podsakoff et al. (2003) argued that CMV is often a problem and that researchers need to take steps to control for this. There are several procedures used to reduce CMV in this research, one of which is to assure respondents of the anonymity and confidentiality of the study. In addition, hiding the meaning of items and adding reversed items in questionnaires may be helpful.

In this research, the meanings of items were invisible, reversed items also added in questionnaires randomly, and respondents assured anonymity and confidentiality. These procedures should decrease respondents’ carelessness, and reduce respondents’ evaluation apprehension and make them less likely to edit their responses to be more socially desirable, lenient, acquiescent, and consistent as to how they think the researcher wants them to respond.

To verify whether the bias of CMV exists, this research first uses Harman’s one-factor test to measure CMV among the variables (Podsakoff et al. 2003). To assess whether there was any evidence of a non-response bias, a comparison between early and late respondents was undertaken following Lindner et al. (2001) proposed that late responders are similar to nonresponders. This allows one to use the late responder group as a surrogate for nonresponde

#### Questionnaire pre-test and revision

This study invited seven experts who were employed by the research object with over 10 years of experience to review and revise the questionnaire item by item so that the questionnaire can have the appropriate content validity. The test standards of content validity are content validity indexes, including the correctness, adequacy, and necessity of the item and the questionnaire overall. The score of the questionnaire tested ranged from 1 to 3. A score of 1 meant that it is inappropriate to use the item and it should be deleted; 2 meant that the item needs to be revised before being used; and 3 meant that it is appropriate to use the item. Items in this study’s questionnaire with a content validity index (CVI) of less than 0.8 should be deleted.

### Methods of data analysis

In order to test the hypotheses, this study use SPSS 20.0 and AMOS 18.0 software as major tools to help us analyze the collected data. To test the hypotheses, the following data analysis methods would be pretested.

- Normality and extreme value testing

Using the AMOS normality and extreme value tests, to understand whether a given sample set of continuous (variable) data could have come from the Gaussian distribution (also called the normal distribution).

b. Questionnaire pre-test

Content validity and Expert Validity were conducted, to test the adequacy of the measurement tool content and evaluate the apparent validity using the judgment method.

c. Descriptive statistic analysis

To better understand the characteristics of each variable, descriptive statistic analysis was used to illustrate the means, and standard deviation of each research variable. Furthermore, to identify the variables that have significant discrepancies for each dimension, an independent *t* test was conducted.

d. Common method variance testing

Harman’s one-factor test and early and late respondent significance test were conducted, to test CMV problem and non-response error.

e. Measurement model testing

A part of the entire structural equation model (SEM) process, which is an analogous to the factor analysis, including all dual items, variables, or observations that “load” onto the latent variable as well as their relationships, variances, and errors.Using both an exploratory factor analysis (EFA) and a confirmatory factor analysis (CFA) to assess construct validity.

f. Structural model testing

First, testing the relationships between different variables. Then, the SEM analysis of the latent variables was conducted, and the empirical analysis of the mediating effects also began with the evaluation of the overall measurement model and then used bootstrapping as the testing method.

The sequence of analysis mentioned above ensured that the measurements were valid and reliable before attempts were made to draw conclusions about the relationships between the constructs. Whether a contribution has been made to the current body of knowledge and whether the research objectives have been achieved will be discussed in the summary.

## Results

### Sample analysis

#### Sample collection

We report on data collected from TWD employees. A total of 350 questionnaires were distributed in total, and 302 were collected for a questionnaire return rate of 86.3 %. After eliminating 27 incomplete questionnaires, the number of valid questionnaires was 275 with a valid questionnaire return rate of 78.6 %.

Before using the AMOS normality and extreme value tests, this study had to ensure that the sample did not have missing values. AMOS’s data imputation functional check confirmed that the sample did not have any missing values. The researchers next conducted normality and extreme value testing. The result of the sample normality is shown in Table 2.

**Table 2:** Result of sample normality

Dimensions	Variable	Min	Max	Skew	Kurtosis	C.R.
Knowledge infrastructure capability	Structure	16.000	40.000	−.268	−.002	
	Information technology	21.000	45.000	−.461	.044	
	Culture	18.000	45.000	−.543	−.214	
Knowledge process capability	Acquisition	17.000	50.000	−.364	−.057	
	Transformation	17.000	50.000	−.781	−.058	
	Application	12.000	30.000	−.355	.137	
Organizational effectiveness	Rational goal	4.000	20.000	−.931	.754	
	Open system	9.000	25.000	−.686	.832	
	Human relationship	8.000	20.000	−.316	−.314	
	Internal process	7.000	20.000	−.244	.673	
Organizational commitment	Affective	9.000	20.000	−.443	.264	
	Normative	9.000	20.000	−.353	.425	
	Continuous	7.000	20.000	−.651	.712	
	Multivariate					1.781

The skew of the sample ranged from −0.931 to −.244, and kurtosis ranged from −.314 to 0.832, meaning that neither exceeded the proposal by Kline (2005) in which the skew acceptable range is 3 or less and the kurtosis acceptable range is 8 or less.

The last line is the C.R. (critical ratio) value of the multivariable (1.781), which did not exceed the suggestion of Li (2011), CR value larger than 2 implies that some variables may have extreme values. Therefore, this study could conduct subsequent statistical analyses of the sample.

#### Questionnaire pre-test

- Content validity

This concept refers to the adequacy of the measurement tool content in terms of inclusiveness and richness. Content validity means the degree to which the questionnaire items can reflect the research topics according to the research framework. This study created the measurement items of the different variables in this study based on previous relevant studies, our revision, and further refinement based on experts’ opinions. These items should demonstrate considerable validity.

(b) Expert validity

This concept refers to experts who were invited to evaluate the apparent validity using the judgment method. This study invited seven experienced professionals employed by the research object who had over 10 years of experience to conduct an expert validity analysis. The experts gave scores according to the adequacy of the items. An item with a score of 1 was considered inadequate, 2 meant adequate after revision, and 3 meant adequate. The CVI was then calculated. The experts’ experience is shown in Table 3, and the CVI of the entire questionnaire is 0.92 (Table 4).

**Table 3:** Years of experience

No.	Position titles	Years of service
1	Specialist of the District Office	30
2	Section Head of the Customer Service Center	20
3	Section Head of the District Office	25
4	Section Head of the District Office	19
5	Section Head of the District Office	17
6	Associate Engineer of District Business	12
7	Officer of the District Office	30

No corresponding relationship exists between experts’ scoring and questionnaire scoring

**Table 4:** Content validity index

Dimensions	CVI
Demographic background	.92
Knowledge management capability	.89
Organizational commitment	.92
Organizational effectiveness	.96
AVG	.92

#### Descriptive statistic analysis

The structured questionnaire used in the study included a section on employee profiles, as various demographic and other factors were likely to influence the organizational effectiveness. Information on demographic features may also be helpful in provide KMC effectively. The demographic variables of the subjects include gender, age, education level, service units, and work seniority. The demographic profile results are shown in Table 5.

**Table 5:** Demographic information of respondents

Measure	Items	Freq.	Percent (%)
Gender	Male	205	74.5
	Female	70	25.5
Age	Older (≧41)	218	79.3
	Younger (<41)	57	20.7
Education level	College (above)	263	95.6
	Other	12	4.4
Service units	Headquarter	81	29.5
	District Business Office	194	70.5
Years of service	Senior (≧11)	213	77.5
	Junior (< 11)	62	22.5

The number of valid respondents in this study is 275. Based on gender, most respondents are male, and the number totaled 205 (74.5 %). The remaining respondents are females, totaling 70 (25.5 %). Based on age, most respondents are 41 and older, totaling 218 (79.3 %). The remaining respondents are under the age of 41, totaling 57 (20.7 %). Based on the level of education, the number of highly educated (college, associate degree’s and above) and basically educated (high school and below) respondents is 263 (95.6 %) and 12 (4.4 %), respectively. Based on the service departments, most respondents are district business officers, totaling 194 (70.5 %). The remaining respondents are at the headquarters, totaling 81 (29.5 %). Based on the service work seniority, most respondents are senior (11 years and above), totaling 213 (77.5 %). The remaining respondents are junior (11 years and below), totaling 62 (22.5 %). There are significant differences between the two groups of respondents, regardless of the demographic profile. The sample condition is consistent with current attributes of the object. Consequently, the random respondents were representative of the larger population.

Furthermore, to identify the variables that have significant discrepancies for each dimension, an independent t test was conducted, and the results are shown in Table 6.

**Table 6:** Significant discrepancy background variables

Dimension	Background variables	Group	Independent *T* test		Compare means	
			*t* value	p value	Mean	SD
Organizational commitment	Age	Younger	−2.293	.024	40.77	7.776
		Older			41.53	6.483
	Education level	High educational level (college, associate’s degree, and above)	−2.147	.034	40.93	6.542
		Low educational level (high school and less)			46.10	7.094
	Work seniority	Junior (11 years and below)	−2.260	.026	40.35	7.346
		Senior (11 years and above)			41.64	6.571

In Table 6, age, education level, and work seniority have significant discrepancies in the organizational commitment dimension. In age variable, the older group, compared to the younger group, reported a higher comparable mean of organizational commitment (41.53 and 40.77 %, respectively). In the education level variable, the low educational level group reported a higher comparable mean of organizational commitment compared to the high educational level group (46.1 and 40.93 %, respectively). In work seniority variable, the senior group reported a higher comparable mean of organizational commitment compared to the junior group (41.64 and 40.35 %, respectively).

#### Common method variance testing

The basic hypothesis of Harman’s one-factor test is that when a main variance exists that can explain most variables’ covariance, this means that a problem of the CMV among variances exists. These technical data load all of the variables into an exploratory factor analysis, and examine the unrotated factor solution to determine the number of factors that are necessary to account for the variance in the variables. As shown in Table 7, both extracted factors have a cumulative variation prediction of 53.799 %. Otherwise, the main factor can only explain 45.303 % of the variance, which indicates that there are no significant underlying dimensions behind all items to prevent a serious CMV problem.

**Table 7:** Harman’s one-factor test

Factor	Extraction sum of squared loading		
	Total	% of variance	Cumulative %
Total variance explained			
1	9.009	45.303	45.303
2	1.104	8.496	53.799
*N* = 275			

Early and late respondents were compared in terms of gender, age, education level, service units and years of service, where early respondents were defined as the first 30 respondents to return the questionnaires and late respondents were the last 30 to return the questionnaires, using traditional t tests following Lindner et al. (2001) recommendations. These data revealed very few significant differences (at the 5 % significance level) between the two groups, thus providing evidence that a non-response error was unlikely to be a major problem in this study (Table 8).

**Table 8:** Early and late respondent significance test

Variable	Items	Early respondents (n = 30)		Late respondents (n = 30)		t	p
		Freq.	Percent (%)	Freq.	Percent (%)		
Gender	Male	22	73.3	23	76.7	.885	.380
	Female	8	26.7	7	23.3		
Age	Older (≧41)	23	76.7	25	83.3	.637	.527
	Younger (< 41)	7	23.3	5	16.7		
Education level	College (above)	28	93.3	29	96.7	.584	.561
	Other	2	6.7	1	3.3		
Service units	Headquarter	8	26.7	10	33.3	.850	.399
	District Business Office	22	73.3	20	66.7		
Years of service	Senior (≧11)	22	73.3	24	80.0	.885	.380
	Junior (< 11)	8	26.7	6	20.0		

### Measurement model testing

A measurement model is a part of the entire structural equation model (SEM) process. This part, which is an analogous to the factor analysis, needs to include all dual items, variables, or observations that “load” onto the latent variable as well as their relationships, variances, and errors.

Past studies (Farrell 2010) suggested using both an exploratory factor analysis (EFA) and a confirmatory factor analysis (CFA) to assess construct validity. An EFA was first conducted to purify the scale and assess the dimensionality of the constructs used.

#### Exploratory factor analysis (EFA)

First, this study conducted an exploratory factor analysis of the concepts in this study. Using the principal component analysis and following Brown (2006), the extracted common factors with eigenvalues larger than 1 and using the varimax orthogonal rotation, this study was able to find that the factor loadings of all of the items were higher than 0.6, as shown in Table 9.

**Table 9:** Measurement model testing results

Dimensions	Variables	Standard factor loading	SMC (R-square)	AVE	Cronbach’s α	
Knowledge infrastructure capability	Structure	.926	.86	.813	.940	.932
	Information technology	.879	.77			
	Culture	.900	.81			
Knowledge process capability	Acquisition	.910	.83	.810	.898	
	Transformation	.845	.71			
	Application	.943	.89			
Organizational commitment	Affective	.897	.81	.772	.907	
	Normative	.879	.77			
	Continuance	.860	.74			
Organizational effectiveness	Rational goal model	.881	.78	.709	.902	
	Open system model	.883	.78			
	Human relationship model	.885	.78			
	Internal process model	.706	.50			

*AVE* average variance extracted, *SMC* squared multiple correlations

Since principal component analysis is the default method of extraction in many popular statistical software packages, including SPSS and SAS, which likely contributes to its popularity. Principal component analysis can produce similar results to true factor analysis when measurement reliability is high and/or the number of factored variables/items increases (Thompson 2004).

It is particularly useful when you need a data reduction procedure that makes no assumptions concerning an underlying causal structure that is responsible for covariation in the data. Because it is a variable reduction procedure, principal component analysis is similar in many respects to exploratory factor analysis.The resulting principal components may then be used in subsequent analyses.

#### Confirmatory factor analysis (CFA)

Reliability, convergent validity, and discriminant validity of the scale were examined using CFA.

- Reliability

Reliability refers to the correctness and precision of a test. The purpose of testing reliability is to verify the correctness or accuracy of the questionnaire. All items within the scale measurement should be internally consistent. The Cronbach’s alphas of the reliability tests in this study are all higher than 0.7, as shown in Table 9. Lance et al. (2006) argued that the lowest acceptable Cronbach’s alpha is 0.7. Hence, the reliability in this study shows fair stability and consistency.

b. Validity

Validity indicates the goodness of fit of the construct with the actual thinking (Neuman 2006). The validity tests the degree to which an instrument measures a particular concept that requires measurement. In other words, validity relates to whether the researchers have measured the right concept as well as the reliability and consistency of the measurement (Hair et al. 2006; Sekaran 2003).

- Convergent validity

Convergent validity can be applied to examine factor loading. All observed item factor loadings for the final measurement model analysis are adequate, ranging from 0.706 to 0.943, as shown in Table 9. The results are above the recommended limit of 0.5 for factor loadings (Hair et al. 2010). The values indicate that every variable was accepted with the convergent validity assessment.

Moreover, the average variance extracted (AVE) values for all constructs exceeded the suggested threshold value of 0.50, thus again demonstrating the convergent validity of the scale (Sekaran 2000) in Table 9.

(b) Discriminant validity

This study used the correlation matrix of the dimensions to test discriminant validity. The square root of the average variances extracted (AVE) from the various dimensions in this study was larger than the correlation between each pair of latent variables. Hence, the discrimination validity was adequate (Hair et al. 2010) (Table 10). Overall, the evidence of reliability and validity indicates the adequacy of testing the relationships between the dimensions at a subsequent stage.

**Table 10:** Result of construct discriminant validity

Dimensions	Knowledge infrastructure capability	Knowledge process capability	Organizational commitment	Organizational effectiveness
Knowledge infrastructure capability	.902			
Knowledge process capability	.705	.900		
Organizational commitment	.695	.679	.879	
Organizational effectiveness	.606	.667	.617	.842

The diagonal line is the square root of AVE, and the other is the correlation coefficient of various dimensions

### Structural model testing (verification of the relationships between the dimensions)

To test the relationships between the different variables, this study first evaluated the structural model. Then, this study conducted an SEM analysis of the latent variables. The empirical analysis of the mediating effects also began with the evaluation of the overall measurement model and then used bootstrapping as the testing method. The structural equation modelling of this study was based covariance.

**Fig. 4:**
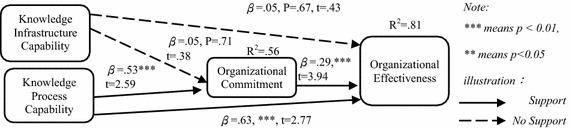
Path analysis of structural model

The results of the path analysis of the structural model in this study are shown in Fig. 4. The model fit index is shown in Table 11, and the results of the structural model are shown in Table 12.

**Table 11:** Model fit index

Index	Range	Goodness of fit standard	Scholar	Outcome	Conclusion
χ^2^/df	The smaller, the better	<3	Kline (2005)	2.59	Hypothesis model fitness
GFI	0–1	>.9	Tabachnick and Fidell (2007)	.93	Model is a good fit
AGFI	0–1	>.9	Tabachnick and Fidell (2007)	.92	Model is a good fit
NFI	0–1	>.9	Kline (2005)	.97	Model is a good fit
CFI	0–1	>.9	Kline (2005)	.98	Model is a good fit
RMSEA	The smaller, the better	<.07	Steiger (2007)	.03	Model is a good fit

*χ* ^2^ */df* Chi squared divided by degrees of freedom, *GFI* Goodness-of-fit Index, *AGFI* adjusted Goodness-of-fit Index, *NFI* Normed Fit Index, *CFI* Comparative Fit Index, *RMSEA* root mean square error of approximation

**Table 12:** Structural model results

Content of hypothesis	Coefficient β	SE	t value	p value	Correlation	Result
H1: KIC has a significant positive effect on organizational effectiveness	.05	.13	.43	.67	.816	Not support
H2: KPC has a significant positive effect on organizational effectiveness	.63	.23	2.77	***	.767	Support
H3: KIC has a significant positive effect on organizational commitment	.05	.13	.38	.71	.695	Not support
H4: KPC has a significant positive effect on organizational commitment	.53	.22	2.59	***	.679	Support
H5: Organizational commitment has a significant positive effect on organizational effectiveness	.29	.07	3.94	***	.717	Support

*** Represents that the correlations are significant at 0.01 or above, ** represents that the correlations are significant at 0.05 or above, * represents that the correlations are significant at 0.1 or above

#### Evaluating the goodness-of-fit criteria

Absolute fit indices determine how well an a priori model fits the sample data (McDonald and Ho 2002) and demonstrates which proposed model has the most superior fit. These measures provide the most fundamental indication of how well the proposed theory fits the data. Included in this category are the Chi Squared, GFI, AGFI, NFI, CFI, and the RMSEA. Because of the problem of the sample size, this study also adopted the suggestion of Kline (2005), that is, χ^2^ divided by the degree of freedom, to eliminate the influence of sample sizes.

As shown in Table 11, χ^2^ divided by the degrees of freedom equals 2.59, which is smaller than 3. The GFI value of 0.93, the AGFI value of 0.92, the NFI value of 0.97, and the CFI value of 0.98 all are higher than or very close to each cut-off value of 0.9. The RMSEA value is just within the acceptable range of 0.07 or less; for this model, the value is 0.03. With these indexes corresponding to the standards, the model is fit to be used in the analysis.

#### Path analysis

Table 12 presents the relationship between KIC/KPC, organizational commitment and organizational effectiveness. The results of this study are summarized as follows:

- Effects of KIC on organizational effectiveness

A relationship between KIC and organizational effectiveness was not found (β = 0.05, t value = 0.43, p value = 0.67); thus, hypothesis 1 is not supported.

b. Effects of KPC on organizational effectiveness

The research showed that KPC had a positive effect on organizational effectiveness (β = 0.63, t value = 3.77, p value < 0.01); thus, hypothesis 2 is supported.

c. Effects of KIC on organizational commitment

A relationship between KIC and organizational commitment was not found (β = 0.05, t value = 0.38, p value = 0.71); thus, hypothesis 3 is not supported.

d. Effects of KPC on organizational commitment

The outcome showed that KPC has a positive effect on organizational commitment (β = 0.33, t value = 2.59, p value < 0.01), thus supporting hypothesis 4.

e. Effects of organizational commitment on organizational effectiveness

As the table mentioned, organizational commitment and organizational effectiveness were related. The research outcome showed that organizational commitment positively affects organizational effectiveness (β = 0.29, t value = 3.94, p value <0.01), thus supporting hypothesis 5.

#### Mediating effects analysis

There are an increasing number of revisions to the current statistical methods that test mediating variables. Compared to the use of regression analysis to test the mediating variable, bootstrapping is an easier analytical method. It is similar to regression analysis; however, when the number of respondents is relatively small, the inferential ability of a regression analysis is relatively insufficient (Preacher and Hayes 2004). Thus, using a regression analysis requires a relatively large number of respondents (Xie 2008).

Bootstrapping revises the regression analysis by testing the effects of the number of respondents and taking such effects into consideration. Thus, even when the sample number is small or the sample shows abnormal distribution, bootstrapping still has an inferential ability (Mattila 2001).

To test the hypothesis that organizational commitment has a significant mediating effect on the impact of KIC and KPC regarding organizational effectiveness (H5a and H5b), this study used the bootstrap in AMOS to test the mediating effects. The results are shown in Table 13.

**Table 13:** Mediating effects of organizational commitment between KIC and KPC on organizational effectiveness

Dimensions	KIC			KPC			Organizational commitment		
	Direct effects	Indirect effects	Total effects	Direct effects	Indirect effects	Total effects	Direct effects	Indirect effects	Total effects
Organizational commitment	.82	…	.82	.03**	…	.03**			
Organizational effectiveness	.81	.78	.78	.06	.02**	.01**	.02**		.02**

*** Signifies that the correlations are significant at 0.01 or above

** signifies that the correlations are significant at 0.05 or above

* Signifies that the correlations are significant at 0.1 or above

This table shows the test result using bootstrapping in which organizational commitment has a significant mediating effect for KPC on organizational effectiveness; thus, hypothesis 5b of this study was supported. In contrast, organizational commitment has no significant mediating effects for KIC on organizational effectiveness, thus not supporting hypothesis 5a.

## Conclusions and suggestions

This study chooses public utility as the research subject. This study therefore contributes to extending the strategy of KMC to the analysis using the employees’ perspective in government organizations. The results of this study provide managers with insights into how to allocate organizational resources and how to improve their organizational effectiveness when deciding on which KMC strategy to adopt. The results of this research can serve as a reference to scholars in this area in the future and can be useful for the management of TWD to understand their organization’s KMC and effectiveness. As a follow-up, research implications and directions are discussed.

### Research conclusions and finding

The major objective of this study is to investigate the interrelationships between KMC and organizational effectiveness. Furthermore, the mediating effects of organizational commitment on the relationships are another objective. Based on the results of this study, several conclusions can be drawn as follows.

First, through a series of statistical analyses based on a survey of 275 sample organizations, several conclusions are made. To examine the effects of the KIC, KPC and organizational effectiveness, this study conducted structural equation modeling (SEM) to test Hypotheses 1–5. Furthermore, to examine the mediating effects of organizational commitment of KIC and KPC on the organizational effectiveness, this study conducted bootstrapping revisions on the regression analysis to test hypotheses 5a and 5b. Thus, even when the sample number is small or the sample shows abnormal distribution, bootstrapping still has inferential abilities (Mattila 2001). The results of the hypotheses in this study are summarized in Table 14.

**Table 14:** Summary of hypotheses in this study

Hypothesis	Content of hypothesis	Support
H1	KIC has a significant positive effect on organizational effectiveness	No
H2	KPC has a significant positive effect on organizational effectiveness	Yes
H3	KIC has a significant positive effect on organizational commitment	No
H4	KPC has a significant positive effect on organizational commitment	Yes
H5	Organizational commitment has a significant positive effect on organizational effectiveness	Yes
H5a	Organizational commitment has a significant mediating effect between KIC and organizational effectiveness	No
H5b	Organizational commitment has a significant mediating effect between KPC and organizational effectiveness	Yes

The results of Table 12 show that the impact of KIC on organizational effectiveness in this study did not reach the significance level, indicating that the hypothesis “KIC has a significant positive effect on organizational effectiveness” (H1) was not confirmed. In other words, hypothesis 1 is not supported. The results in this table also show that the effect of KPC on organizational effectiveness in this study reached the significance level, showing that the hypothesis “KPC has a significant positive effect on organizational effectiveness” (H2) was confirmed. In other words, this hypothesis is supported. As the table above shows, the effect of KIC on organizational commitment in this study did not reach the significance level, indicating that the hypothesis “KIC has a significant positive effect on organizational commitment” (H3) was not confirmed. In other words, this hypothesis is not supported. The results in this table also show that the effect of KPC on organizational commitment in this study reached the significance level, showing that the hypothesis “KPC has a significant positive effect on organizational commitment” (H4) was confirmed. In other words, this hypothesis is supported. The results of Table 12 show that the effect of organizational commitment on organizational effectiveness in this study reached the significance level, showing that the hypothesis “organizational commitment has a significant positive effect on organizational effectiveness” (H5) was confirmed. In other words, this hypothesis is supported.

The results of Table 13 show that the direct effects and total effects of KIC on organizational commitment in this study are insignificant; the direct effects, indirect effects and total effects of KIC on organizational effectiveness in this study are insignificant. Based on the foregoing data, the hypothesis “organizational commitment has significant mediating effects between KIC and organizational effectiveness” (H5a) was not confirmed. In other words, this hypothesis is not supported. The results of this table also show that the direct effects and total effects of KPC on organizational commitment in this study are significant; the direct effects, indirect effects and total effects of KPC on organizational effectiveness in this study are significant. Based on the foregoing data, the hypothesis “organizational commitment has significant mediating effects between KPC and organizational effectiveness” (H5b) was confirmed. In other words, this hypothesis is supported.

To summarize, first, regardless of the relationships between KIC and organizational effectiveness or the relationships between KIC and organizational commitment, no significant effects were found. The relationships between KPC and organizational effectiveness and the relationships between KPC and organizational commitment have significant effects. Meanwhile, KPC, through organizational commitment, positively influences organizational effectiveness. Our findings confirm that KMC is not solely sufficient to drive organizational effectiveness and that organizations also need to encourage organizational commitment.

Second, there are significant differences between the two groups of respondents, regardless of the demographic profile. In this study, age, education level, and work seniority have significant discrepancies on organizational commitment. The results imply that the older, lower educated, senior groups of respondents have higher organizational commitment levels than younger, higher educated, junior respondent groups.

### Theoretical implications

This paper represents one of the earliest studies that analyze the use of organizational commitment for KMC in organizational effectiveness. This article developed and tested a model to explain the effects of KIC and KPC on organizational effectiveness and considered the mediating role played by organizational commitment on the organizational effectiveness. This study made certain significant contributions to the foregoing literature in a number of ways. Thus, the empirical findings complement and extend the previous research.

First, in terms of the research object selection, past KMC-related studies chose cases from finance and manufacturing firms (Gold et al. 2001), manufacturing and service organizations (De Long and Fahey 2000), and manufacturing firms (Shu 2004). As this paper mentioned previously, most of the relevant previous studies generally used private firms as their research subjects. This study chose a public utility company as its research object, hoping to expand the scope of relevant studies on KMC, organizational effectiveness, and organizational commitment to fill this important gap and serve as a reference to scholars in this area in the future.

Second, with regard to population selection, a rich selection of literature was examined with KMC with subjects such as chief knowledge officers (De Long and Fahey 2000), senior executives (Gold et al. 2001), practitioners and researchers (Holsapple and Joshi 2002), professionals (Khalifa and Liu 2003), middle managers (Lee and Choi 2003), and R& D managers (Shu 2004). The sampled population of this study was the staff of the Taipei Water Department because the labor at TWD was almost entirely outsourced. Staff members become knowledge workers who disseminate information throughout communities and find or provide a way to address problems. To be successful at KM, particularly in providing services to the public, all staff members should be responsible for managing all types of knowledge that are available in the organization.

Third, this study presents a hypothesized model that shows not only the correlation of KMC and organizational effectiveness but also presents the mediator role of organizational commitment. It adds new knowledge to management science on several fronts relating KMC, namely providing an in-depth look at organizational commitment, KMC and organizational effectiveness as related in a public utility company and demonstrates a clear path to organizational effectiveness for future research.

Fourth, surprisingly, this research shows no significant relationship between KIC and organizational effectiveness. As this paper mentioned previously, these results are different from the conclusions of De Long and Fahey (2000), Holsapple and Joshi (2002), Gold et al. (2001), and Lee and Choi (2003) that the related literature and previous research findings. So as only organizational commitment has significant mediating effects between KPC and organizational effectiveness, whereas this is not the case for KIC and organizational effectiveness.

A possible reason for these results is that TWD has developed KM for a long period of time, and the related knowledge infrastructure may be quite complex. Therefore, the employees’ perspectives of KIC have not been very strong. Another reason is that social media tools on the internet now drive more powerful forms of collaboration. Knowledge workers engage in “peer-to-peer” knowledge sharing across organizational and company boundaries, forming networks of expertise (Tapscott and Williams 2006).

The relationships between KPC and organizational effectiveness show a significant relationship. As expected, the results of this study are consistent with the views of previous studies and literature.

### Practical implications

Practically, this study is the first formal study evaluating KMC in a benchmark water utility company in Taiwan. The results of the proposed study will assist managers by pointing out areas of strength and highlighting the perception of organizational effectiveness and organizational commitment. By focusing on these findings, managers can develop and enhance organizational effectiveness, thereby establishing and maintaining the long-term KIC and KPC strategy of an organizational direction, such as Taiwan. As this paper mentioned previously, Alavi and Leidner (2001) believed that KM aims at building organizational competencies, understanding strategic know-how, and creating intellectual capital when knowledge is considered from a capability perspective. Mouritsen and Larsen (2005) argued that the second wave of KM concerns the viewpoint of knowledge resources and organizational competencies.

Second, this study identified another element that is also important for any public sector organization, namely organizational commitment. The authors believe that it is very important to manage this dimension accordingly, especially if the government wants to implement a knowledge management strategy in a public organization, because knowledge transfer requires the willingness of a group or individual to work with others and share knowledge to their mutual benefit. Without sharing, it is almost impossible for knowledge to be transferred to others. This shows that knowledge transfer will not occur in an organization unless its employees and work groups display a high level of cooperative behavior (Goh 2002). A worker’s performance is greatly dependent on their motivation, inspiring them to come to work regularly, work diligently, be flexible, and willing to carry out their duties (Ashraf et al. 2014; Kok et al. 2015). Organizational development does indeed focus on enabling a change in organizational culture, attitudes, values, and beliefs, which emphasize and support healthy processes and interpersonal relations at work (Hodgins et al. 2014). If all of the dimensions (KIC, KPC, and organizational commitment) can be managed efficiently and effectively, knowledge can be easily created and transferred in the organization.

Third, knowledge workers are represented by subject-matter specialists in all areas of an organization, and social media networks enable knowledge organizations to co-produce knowledge outputs by leveraging their internal capacity with massive social networks. Human Interaction Management (Harrison-Broninski 2005) asserts that there are five principles characterizing effective knowledge work: (1) Build effective teams; (2) Communicate in a structured way; (3) Create, share and maintain knowledge; (4) Align your time with strategic goals; and (5) Negotiate next steps as you work. If the knowledge can be retained, knowledge worker contributions will serve to expand the knowledge assets of an organization. In particular he/she, through his/her workspace, is able to browse the organizational Knowledge Base to get support in an unexpected (that is, not defined in advance) way, so discovering useful, hidden connections (Nunes et al. 2014). Competences within the organization are developed through several channels, and organizations need the constant and usable availability of learning resources from different devices; in fact 51 % of the learning resources are unstructured, namely received outside of canonical training activities (Aberdeen Group 2014). Furthermore, employees need to have at their disposal tools that improve their capacity to share knowledge with colleagues wherever and whenever. These technologies enhance knowledge management and usually involve more people in knowledge creation process as they allow multiple people to collaborate when creating knowledge (Majchrzak et al. 2013).

The above mentioned about the findings show that different from earlier findings in the literature, that is to say neither KIC and organizational effectiveness are insignificant, nor organizational commitment has insignificant mediating effects between KIC and organizational effectiveness. It is noteworthy that the above findings inspired managers, according to Tapscott and Williams (2006) suggestion, in addition to construct the knowledge infrastructure more than focus on social media tools on the Internet, which engage knowledge workers in “peer-to-peer” knowledge sharing across organizational and company boundaries.

Fourth, the results in this research can be the benchmark of operations for domestic knowledge-based public utility companies. As this paper mentioned previously, De Angelis (2013) state the public sector is influenced by a growing need for: “competition, performance standards, monitoring, measurement, flexibility, emphasis on results, customer focus and social control”. This study should help managers understand the inter-relationship between the KMC and organizational commitment as the mechanism for enhancing organizational effectiveness. Understanding the current situation and actual needs of employees can help organization key success factors, improve overall competitiveness and operational performance, and upgrade managerial standards.

### Research limitations

This study, like all other studies, suffered various limitations that restrict the generalization of the findings and opens directions for future research. Even though this study attempts to be as rigorous and objective as possible, the following limitations remain based on the literature review, research methods, data collection, and statistical analyses.

First, because this study only focused on one public utility (tap water) in a specific country (Taiwan), the findings cannot be generalized to other service sectors and different geographical areas. Meanwhile, the sampled populations of this study were the staff of the Taipei Water Department, and the characteristics of the sample are unlikely to be seen in other areas. Therefore, the results of the statistical analyses cannot be applied to other organizations in Taiwan.

Second, this study distributed questionnaires to verify the hypotheses, which is a temporal cross-sectional approach, and the samples were still material from the same period. Theoretically, conducting a longitudinal study to collect data can better support causal inference (Beugre and Viswanathan 2006). Therefore, the causal inference in this study seems slightly weak.

Third, this study only explores the mediator role of organizational commitment without considering other factors. As a consequence, this research fails to enumerate all of the potential factors of all the mediator roles of KMC with organizational effectiveness.

### Suggestions for future studies

Although the result of this study may contribute to verifying the phenomenon in Taiwan, several suggestions could be made for academicians and business practitioners.

First, the study exposed a number of opportunities for further examination pertaining to organizational elements that influence the success of implementing knowledge management as a whole. One of the elements that need further research is the knowledge infrastructure capability (KIC); research in this area, particularly in a private or public organization, could have different results. Another important area that needs to be explored more is organizational commitment. We believe that the success of implementing knowledge management in a public organization will be in line with this area.

Second, in the meantime, although this research cannot take into account all of the correlations of KMC and organizational effectiveness in other public utility fields and even private organizations, the overall structure and process can be employed in an analysis and discussion in other areas.

Third, this study used a convenience sampling method consisting of 275 responses. Future research can overcome this limitation by taking a larger, randomly-selected sample, which may provide a more comprehensive result. Subsequent studies can attempt to apply a qualitative research method and conduct in-depth and long-term studies of specific service providers or use the interview method and conduct face-to-face interviews with the expectation that these methods may obtain data that are more relevant. Addressing qualitative research methods was beyond the scope of this research, and we invite future research to shed additional light on these important issues.

Furthermore, this study uses single informant reports for the variables included in the models, indicating the possibility of a common method bias. Because this study focuses on a rather narrow issue concerning KMC and organizational effectiveness and the informants were well-qualified to report on the variables, this weakness should be able to be mitigated. To ensure that the common method bias is not a problem and to generalize these research findings to other sectors and different geographical areas, future research can replicate this study in other sectors and different countries to overcome the limitations. In addition, this study suggests that scholars can conduct cross-regional comparative studies to expand the research scope in the future. Such research results will help expand the breadth of research and serve as a significant reference for managers who are preparing cross-regional KMC strategies.

Finally, based on this study’s limitations, future research may consider some other mediating variables in the relationship between KMC and organizational effectiveness. For example, future studies can consider including environmental variables (e.g., media effects) and enabling factors of knowledge workers (e.g., human resource policies) into their questionnaires to rigorously test the effects of environmental variables or human resources on organizational effectiveness and increase the richness of the research model content.
